# Model-based peak alignment of metabolomic profiling from comprehensive two-dimensional gas chromatography mass spectrometry

**DOI:** 10.1186/1471-2105-13-27

**Published:** 2012-02-08

**Authors:** Jaesik Jeong, Xue Shi, Xiang Zhang, Seongho Kim, Changyu Shen

**Affiliations:** 1 Department of Biostatistics, Indiana University, 410 West 10th Street, Indianapolis, IN 46202, USA; 2 Department of Chemistry, University of Louisville, 2320 South Brook Street, Louisville, KY 40292, USA; 3 Department of Bioinformatics and Biostatistics, University of Louisville, 485 E. Gray St, Louisville, KY 40292, USA

## Abstract

**Background:**

Comprehensive two-dimensional gas chromatography time-of-flight mass spectrometry (GCxGC/TOF-MS) has been used for metabolite profiling in metabolomics. However, there is still much experimental variation to be controlled including both within-experiment and between-experiment variation. For efficient analysis, an ideal peak alignment method to deal with such variations is in great need.

**Results:**

Using experimental data of a mixture of metabolite standards, we demonstrated that our method has better performance than other existing method which is not model-based. We then applied our method to the data generated from the plasma of a rat, which also demonstrates good performance of our model.

**Conclusions:**

We developed a model-based peak alignment method to process both homogeneous and heterogeneous experimental data. The unique feature of our method is the only model-based peak alignment method coupled with metabolite identification in an unified framework. Through the comparison with other existing method, we demonstrated that our method has better performance. Data are available at http://stage.louisville.edu/faculty/x0zhan17/software/software-development/mspa. The R source codes are available at http://www.biostat.iupui.edu/~ChangyuShen/CodesPeakAlignment.zip.

**Trial Registration:**

2136949528613691

## Background

Comprehensive two-dimensional gas chromatography time-of-flight mass spectrometry (GCxGC/TOF-MS) has been employed in analysis of complex samples in metabolomics studies, especially in cancer [1-3]. However, experiment output still suffers from substantial variations, challenging the data interpretation in these studies. For this reason, the methodological development at the data analysis stage has become a crucial research area, which is still at its infancy. Throughout the paper, we mean experiment by technical run, which is performed by machine.

In practice, there are two types of experiment variations: variation within an experiment and variation between experiments. The need to control variation within an experiment has been reported by many researchers [4-6]. Theoretically, all instrument signals generated by one metabolite species should be reported as a single peak within an experiment. However, in reality, multiple peak entries can occur due to the abnormality in peak shape, high sensitivity of the peak detection algorithm, and experimental cause such as short modulation time [4-6]. To reduce such variation, peak merging should be done before proceeding to any subsequent analysis. On the other hand, variation between experimental runs is induced by factors such as difference in experiment configuration and run-to-run variations. Between-experiment variation usually is much higher in magnitude than within-experiment variation. Specifically, retention time (RT) depends heavily on experimental setup by the nature of experiment. For analysis purpose, different retention times of the same metabolite between experiments should be adjusted for further analysis. The process of such an alignment is usually referred to as peak alignment. Typically, a peak alignment process includes two steps: peak matching where the identity of peaks from each experiment is matched, and RT adjustment that is based on the results of the peak matching.

Several studies addressed the alignment issue of metabolomic profiling from GCxGC/TOF-MS experiment [4-9]. From a methodological perspective, we can classify them into three categories. In the first generation methods [7-9], alignment implies RT adjustment, which is solely based on data of the retention time without the input of metabolite identification. For example, algorithms for aligning local region of interest were introduced in Fraga *et al*. and Mispelaar *et al*. [7,8]. And then, algorithm to align entire chromatogram in two-dimensional GC was suggested by Pierce *et al*. [9]. However, the limitation of those methods is that aligning metabolites by using two-dimensional retention times only may produce false alignment because some metabolites with similar chemical functional groups have similar retention times in both GC dimensions [5]. For this reason, the second generation methods have been developed exploiting two different types of information: closeness in two-dimensional retention times and spectrum similarity [4-6]. Three well-known methods of this generation are MSort [4], DISCO [5], and mSPA [6]. Since DISCO is a modified version of MSort, they are similar in many respects. Thus, our focus is on the difference between mSPA and the other two. First of all, the process of peak alignment in mSPA includes metabolite matching only, without reference to RT adjustment while the other two address both. Second, Kim *et al*. [6] defined and used a mixture similarity score, weighted average of RT distance and spectra similarity for peak matching while the other two used both information sequentially in each step. Third, as a spectrum similarity measure, Kim *et al*. [6] used dot product and the other two used Pearson's correlation coefficient. More comparison among these three methods can be found in [6].

Our method, as a third generation method, is unique in that it is model-based approach. Compared to the second generation methods, our method is different in many respects. Compared to mSPA, our method considers rank distance of retention time instead of Euclidean distance which is used in mSPA. As a spectrum similarity measure, we use cosine score, angle between two spectra while mSPA uses dot product which is the cosine value of the angle. Also, our method covers both homogeneous and heterogeneous data while mSPA can handle homogeneous data only. For clarity, when we get data under the exactly same experiment configuration, we call it homogeneous. Otherwise, we call it heterogeneous. Most of all, our method uses posterior probability for metabolite matching based on an empirical Bayes model. The mSPA, however, defines an ad hoc likelihood function and maximises the function. Compared with DISCO, there are some aspects in common: both methods (1) can be applied to homogeneous and heterogeneous data, (2) address both peak matching and RT adjustment. On the other hand, they differ in four key ways: (1) our method does not need any RT transformation at the pairwise peak matching stage, (2) we use posterior probability as a matching confidence, (3) we use lattice-wise method for RT adjustment, not peak-wise, (4) as a similarity measure, we use mixture similarity score with cosine score involved, but DISCO use Pearson's correlation coefficient.

Since DISCO can be applied to heterogeneous as well as homogeneous data, we compare our method with DISCO. In what follows, we provide a brief description of the model. Then we demonstrate the performance of our method with a mixture of standard compounds and a rat plasma data.

## Results

### Experiment datasets

Two different types of experiments are analyzed in this study: a mixture of standard compounds (Experiment I) and a rat plasma (Experiment II). We have three sets of homogeneous data from Experiment I corresponding to three different temperature gradients, respectively: dataset1 (5°C/min) with 10 replicates, dataset2 (7°C/min) with 2 replicates and dataset3 (10°C/min) with 4 replicates. To produce a heterogeneous dataset by using three homogeneous datasets available, we selected one technical replicate from each dataset and combined them, which is called dataset4. Thus, we have 10, 2, 4, 3 technical replicates in each dataset from Experiment I. From Experiment II, we have 5 homogeneous technical replicates but, no heterogeneous output. More details of experiments are given in Additional file 1.

### Overview of algorithm

To help understanding of our results, we summarize our algorithm briefly because the order of results follows that of our algorithm. After peak merging, we select two experiment outputs and calculate matching confidence of peaks in the form of posterior probability through the empirical Bayes model. Based on these matching confidence (Equation 8 in Methods section), we select metabolite pairs with high matching confidence by applying cutoff value to the posterior probability. We then continue the same pair-wise process for all other experiment outputs and generate representative landmark peaks. Given landmark peaks, we adjust RT of all peaks with respect to these peaks.

### Peak merging

Peak merging is performed based on the result obtained by ChromaTOF software. In the case that multiple peaks exist, we select the peak with maximum peak area and remove the others [6]. The number of compounds before and after peak merging is summarized in Tables 1 and 2.

**Table 1:** Summary of Experiment I: number of compounds after/before peak merging

Run ID N	*R*_1_^5^* 78/183	*R*_2_^5^ 76/188	*R*_3_^5^ 76/163	*R*_4_^5^ 75/152	*R*_5_^5^ 74/154	*R*_6_^5^ 73/147
Run ID N	*R*_7_^5^ 74/175	*R*_8_^5^ 76/164	*R*_9_^5^ 77/171	*R*_10_^5^ 75/175	*R*_1_^7^* 75/134	*R*_2_^7^ 73/171

Run ID N	*R*_1_^10^* 76/150	*R*_2_^10^ 73/139	*R*_3_^10^ 76/114	*R*_4_^10^ 75/119		

n1/n2 presents the number of compounds after/before peak merging; Regarding *R*_*a*_^*b*^, *a *and *b *present experimental replicates and gradient temperature, respectively. Heterogeneous data (Dataset4) are composed of three experiment outputs denoted by *

**Table 2:** Summary of Experiment II: number of compounds after/before peak merging

Run ID N	*D*_1_ 466/759	*D*_2_ 456/733	*D*_3_ 437/695	*D*_4_ 452/727	*D*_5_ 418/661

n1/n2 presents the number of compounds after/before peak merging

### Landmark peaks

Here we choose threshold value (*h *= 40) which is used to calculate *a_j, _b_j _*and b_j_* in layer 2 of our model (see Methods section). Also, we use weight (*w *= 0.1) for mixture score and apply cutoff value of 0.9 to matching confidence, posterior probability of correct match for landmark peak selection.

In Experiment I, 11, 40, 28 and 24 landmark peaks were selected for each dataset, respectively. In Experiment II, 31 landmark peaks were selected.

### Peak alignment results

**Figure 1:**
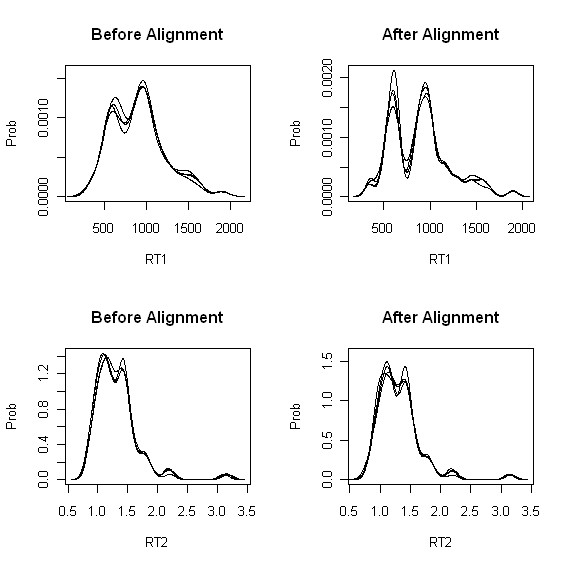
**Experiment I: KDE plots before/after peak alignment for homogeneous experiment (dataset3)**. Top: kernel density estimate (KDE) corresponding to the first retention time before (left)/after (right) peak alignment. Bottom: kernel density estimate of the second retention time before (left)/after (right) peak alignment.

As an efficient way to illustrate alignment results in each dimension of RT (say marginal view), we consider kernel density estimate (KDE) along with normal kernel, which can be considered as a continuous version of histogram. Each KDE plot was made by using retention times of peaks to show the density of retention time. The brief introduction of KDE is given in Additional file 1 (see Section 1-4). KDE plots before/after RT adjustment for homogeneous data (dataset3) are given in Figure 1. Kernel density estimates corresponding to the first retention time before (left)/after (right) RT adjustment are provided in the top row and those corresponding to the second retention time are provided in the bottom row. Based on the KDE plots for homogeneous data, it is clear that after RT adjustment modes from 4 curves are well overlapped and are sharper. In other words, there are more densities around the mode and more distinct hills and valleys after adjustment, implying that peaks are well aligned.

**Figure 2:**
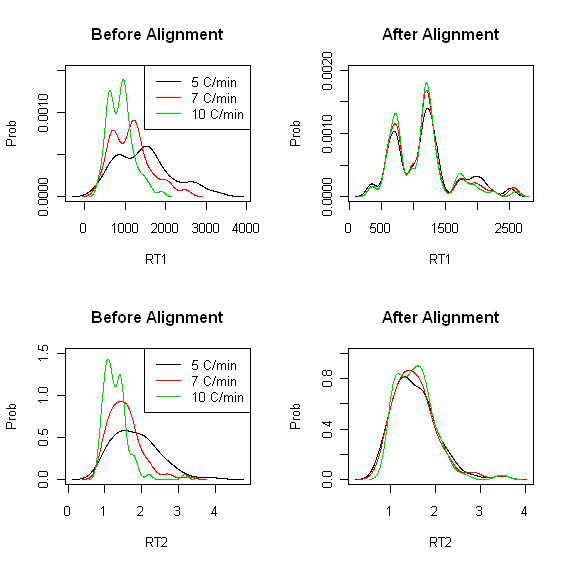
**Experiment I: KDE plots before/after peak alignment for heterogeneous experiment (dataset4)**. Top: kernel density estimate (KDE) corresponding to the first retention time before (left)/after (right) peak alignment. Bottom: kernel density estimate of the second retention time before (left)/after (right) peak alignment.

Similarly, KDE plots for heterogeneous case (dataset4) before/after RT adjustment are given in Figure 2. As expected, there are more run-to-run variations in heterogeneous data than homogeneous ones and after RT adjustment we can see much more dramatic change in heterogeneous case. It is clear that the different RT ranges (presented by different colors in Figure 2) are well adjusted after peak alignment.

**Figure 3:**
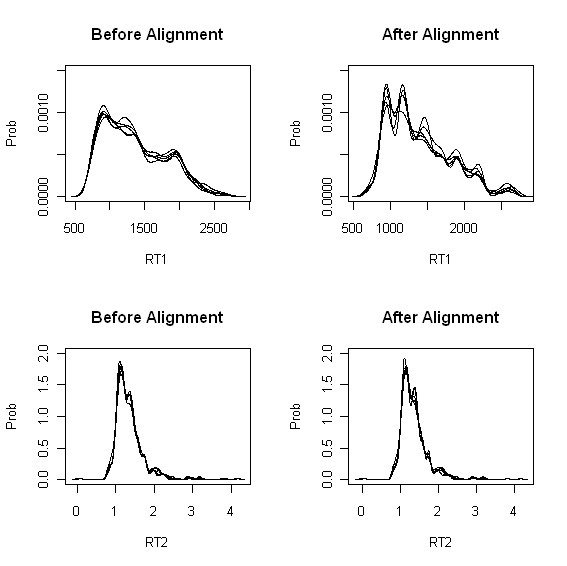
**Experiment II: KDE plots before/after peak alignment for rat plasma data**. Top: kernel density estimate (KDE) of the first retention time before (left)/after (right) peak alignment. Bottom: kernel density estimate of the second retention time before (left)/after (right) peak alignment.

The four KDE plots for more complicated biological sample (Experiment II) are given in Figure 3. We see the same situation as seen in standard mixture data, i.e., sharper and distinct hills and valleys after alignment.

**Figure 4:**
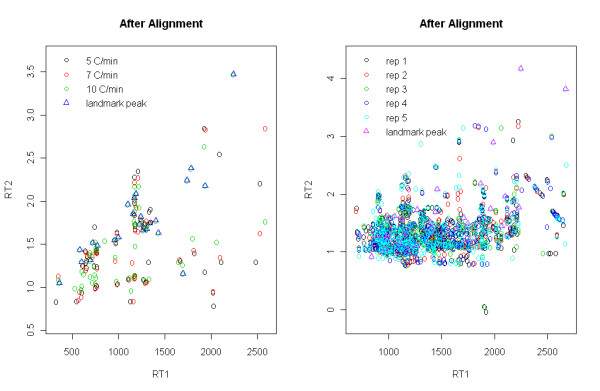
**Two dimensional view on alignment results**. Scatter plots after peak alignment for dataset4 (left) and rat plasma (right).

For two dimensional view on alignment results (say joint view), scatter plots of RT after RT adjustment corresponding to KDE plots in Figures 2 and 3 are given in Figure 4. We can see after alignment that peaks are superimposed very well, implying that peaks with similar retention times are well aligned. More results for other datasets are provided in Additional file 1.

### Performance comparison

For comparison, we select an existing peak alignment method which is able to align both homogeneous and heterogeneous data, DISCO [5]. As a comparison measure, we consider F1 score, with higher value implying better performance. F1 score is calculated under the assumption that ChromaTOF identification results are gold standard, harmonic mean of positive predictive value (PPV) and sensitivity [6,10]. For our method, we considered four different weights (*w *= 0.1, 0.2, 0.3, and 0.5) and 5 different cutoff values (0.6, 0.7, 0.8, 0.9, and 0.95) for posterior probability calculation. For each combination of *w *and cutoff values, we calculated F1 score for each paired technical runs within each dataset. For instance, given a pair of experimental outputs, we get 20 F1 scores, i.e., each score is calculated with each parameter combination, respectively. Among them, we selected the best F1 score. Then, average of such best F1 values obtained from all pairs was calculated. Once it is done, we repeat the same calculation for each dataset: dataset1,..., dataset4 and rat plasma data. Results corresponding to dataset1, dataset4 and rat plasma are summarized in Table 3. More results are provided in Additional file 1.

**Table 3:** Averaged best F1 score: our method v.s. DISCO

Method	Homogeneous	Heterogeneous	Rat
DISCO	0.902	0.781	0.496
Our	0.878	0.839	0.567

The best F1 score for each pair is calculated and then averages of such best F1 values obtained from all pairs for each dataset are calculated. Homogeneous and heterogeneous mean dataset1 and dataset4 from Experiment I, respectively. Rat means Experiment II

Based on the results, it is clear that the performance of our method is better than DISCO except homogeneous case from Experiment I. As seen in the results, our method has better performance for complex data. More precisely, the difference in performance is getting bigger as the complexity of the data increases.

**Figure 5:**
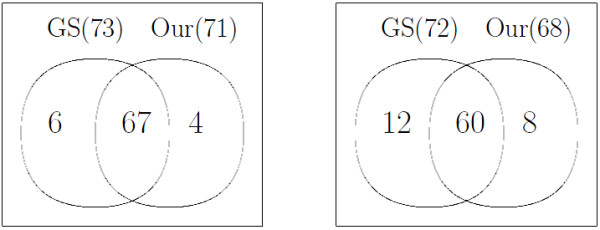
**Experiment I: Venn diagram corresponding to best F1 measure for homogeneous(left), heterogeneous(right) data**. R_8_^5 ^and R_9_^5 ^(*w *= 0.2 and cutoff = 0.8) were selected for homogeneous case and *R*_1_^5 ^and *R*_1_^7 ^(*w *= 0.1 and cutoff = 0.6) were selected for heterogeneous case. The best F1 scores for each case are 0.93 and 0.86, respectively. GS stands for gold standard.

We investigated the relationship between alignment results by gold standard and our method. For the purpose of comparison, we assumed that the identification by ChromaTOF is correct and then aligned the peaks based on their assigned names, resulting in the alignment by gold standard (GS). However, there might exist false positives in the aligned peak list of the gold standard if ChromaTOF assigned a wrong name to a compound. Actually, it is known that the accuracy of ChromaTOF identification is about 80%. To examine the concordance in the results of both methods, three sets of experimental pairs with the best F1 score (i.e., each pair come from each of three different datasets respectively) were selected. In Experiment I, *R*_8_^5 ^and *R*_9_^5 ^(*w *= 0.2 and cutoff = 0.8) were selected for homogeneous case (F1 = 0.93) and *R*_1_^5 ^and *R*_1_^7 ^(*w *= 0.1 and cutoff = 0.6) were selected for heterogeneous case (F1 = 0.86). Numbers in parenthesis in Figure 5 represent the number of compound pairs matched by each method in Experiment I. In the case of homogeneous data (left panel in Figure 5), our method found 71 peak pairs and 67 pairs of them had the same compound name identified by ChromaTOF. On the other hand, for heterogeneous case (right panel in Figure 5), our method found 68 matching pairs and 60 of them were verified to have same compound name by ChromaTOF.

**Figure 6:**
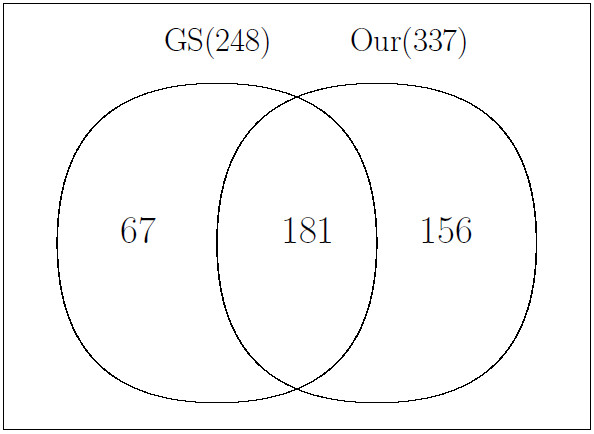
**Experiment II: Venn diagram corresponding to best F1 score for rat plasma experiment**. *D*_3 _and *D*_4 _(*w *= 0.1 and cutoff = 0.8) were used. GS stands for gold standard.

Similarly, Venn diagrams corresponding to the best F1 score for Experiment II (rat plasma) are given in Figure 6. *D*_3 _and *D*_4 _(*w *= 0.1 and cutoff = 0.8) were selected (F1 = 0.62). Our method found 337 peak pairs and 181 pairs of them had the same compound name identified by ChromaTOF. More results for other datasets are provided in Additional file 1.

### Manual validation

To investigate the discordance in the results of both methods, we manually checked the alignment results for homogeneous data from Experiment I (left panel in Figure 5). Since 67 common peak pairs were aligned by both methods, our focus was on the other parts. The four peaks aligned by our method, which have different names given by ChromaTOF, were further analyzed based on raw image data. It is clear that one of them is correctly aligned. However, the other three might be incorrect. In addition to that, six peak pairs which had the same compound name by ChromaTOF, but not aligned by our method were also manually examined. Among them, ChromaTOF made wrong decision for three of them while the other three are correct.

To further investigate the discordant results, we selected two peaks (CAS 193-39-5 and 629-92-5) in target. They were aligned by both method but, aligned to different compounds in library (denoted by * in Additional file 1, Table S8). Those peak pairs aligned by GS were not the best peak pairs by our method, but the second best. For example, a compound with CAS(193-39-5) in target peak list was aligned to the compound with CAS(191-24-2) in reference peak list by our method, not to the compound with CAS(193-39-5) because peak pair (193-39-5 and 191-24-2) had a similarity score of 9.67 while peak pair (193-39-5 and 193-39-5) had a similarity score of 9.69 (i.e., in our method, a pair with the smaller similarity score is selected). Similarly, even though the compound (CAS 629-92-5) was assigned to different compound in library, the difference in score was not substantial. More details about the four and six peaks aligned by each method only are summarized in Additional file 1 (see Section 4.5).

## Discussion

Compared to the existing peak alignment method DISCO that can also be applied to heterogeneous as well as homogeneous data, our method has many unique properties. First, our method is a model-based approach. Second, unlike the DISCO, our method does not need any type of data transformation such as z-score transformation at any stage of the process. For example, in case of heterogeneous data, DISCO first transforms retention times using z-scores in order to reduce the retention time variation among different GCxGC-MS experiments. In other words, data transformation converts heterogeneous data into (pseudo) homogeneous data. Although a well-defined transformation will definitely improve the peak alignment (especially, for heterogeneous data) through the reduction of false positives caused by retention time variation, it is not easy to find an optimal transformation function that can handle all kinds of variation. Therefore, requirement of transformation is usually considered as a downside and it is avoided. Third, once representative landmark peaks are obtained, we make grid net using their retention times and adjust retention times of all peaks with respect to the grid net sequentially in both dimensions (all peaks simultaneously in each dimension).

With a mixture of metabolite standards and a rat plasma data, it is shown that our method has good performance in terms of F1 score. The F1 score requires gold standard (GS) and we used ChromaTOF identification results as a tentative gold standard. In the case of mixture of metabolite standards, even though more variations are involved in heterogeneous data, F1 score is always good regardless of data type. On the other hand, F1 score for complicated data from rat plasma is not that much high even though it is homogeneous. Compared to standard mixture data, although we see some decrease in F1 value for complicated data, the value from our method is still higher than that of the other existing method.

We compared our matching results with the tentative GS. Through the comparison, even though there is some discordance, we see high level of concordance in matching results by both methods, resulting in high F1 score for our method.

## Conclusion

In this paper, we developed a model-based peak alignment method to handle experiment variations, which can be applied to both homogeneous and heterogeneous experiments. Our method utilises a part of the output of ChromaTOF software as input data. The workflow of the method consists of two steps: pairwise peak matching and retention time adjustment. Due to the use of landmark peak lists composed of peak pairs with high matching confidence, our approach produces good quality of peak alignment.

In the peak alignment context, the excellent performance of our method at the data processing stage will have an enormous positive effect on subsequent analysis. For example, even though experiments are performed under the different experiment configurations or even at different times, the data aligned by our method can be used as input for further analysis: for example, time course metabolomic data analysis. Thus, the area to which our method can be applied might be extended to metabolite biomarker finding and metabolite clustering. Furthermore, as a future study, we we will study the relationship between peak alignment and peak identification to improve the accuracy of both preprocessing.

## Methods

Our empirical Bayes model for pairwise peak matching between technical runs utilises the same structural hierarchy as the model constructed for metabolite identifications in [11]. Here we briefly review the model. Suppose that we have two experiment outputs and consider one of them as reference and the other as target in the context of peak alignment.

### Model Review

We consider a hierarchical statistical model with four layers. All layers together address the process of our algorithm. Here is a brief overview of our hierarchical model: (i) we first check if a compound in library is in sample, (ii) depending on the information given in (i), we check if the compound is matched to any compound in sample, (iii) we then check if the match is correct because our matching using similarity score is not always correct, (iv) we finally estimate the distribution of similarity score.

**Layer 1: **We consider the marginal probability that each metabolite in the reference is present in target:

(1) $$\text{P}\left(Y_{j}=1\right)=\rho ,\;j=1,2,\cdots \;,N,$$

where *N *is the number of the peaks in the reference.

**Layer 2: **Given the *Y_j _*information, we consider the conditional probability of metabolite *j *being matched to some target metabolite. According to the value of *Y_j_*, two different conditional probabilities are considered: P[*Z_j _*= 1|*Y_j _*= 0] and P[*Z_j _*= 1|*Y_j _*= 1]. Note that even though a metabolite *j *does not exist in target (*Y_j _*= 0), there is some chance for the metabolite to be claimed as present (P[*Z_j _*= 1|*Y_j _*= 0] > 0). For the case *Y_j _*= 0, we consider the following model:

(2) $$\text{P}\left[Z_{j}=1|Y_{j}=0\right]={\eta_{0}}^{I\left(b_{j}=0\right)}\gamma {\left(\beta ;b_{j}\right)}^{I\left(b_{j}>0\right)},$$

Where $\gamma \left(\beta ;b_{j}\right)=1-\frac{1}{1+\exp\left(\beta_{0}+\beta_{1}b_{j}+\beta_{2}{b_{j}}^{2}\right)}$and *η_0 _*is an unknown constant. *η*_0 _is a kind of auxiliary parameter which is not of our interest because it is related to metabolites with no matching neighbor. The *b_j _*is defined using the metabolite in reference:

(3) $$b_{j}=\underset{k\neq j,k\in C,I\left(r_{kj}<h\right)}{\sum }1/ak,$$

where $a_{k}=\sum_{q\in C}I\left(r_{qk}<h\right),r_{qk}$is a mixture similarity score between peaks *q *and *k *in the reference, *C *is the set of peaks in the reference, and *I*(·) is the indicator function.

Similarly, we consider the following model for the case *Y_j _*= 1:

(4) $$\text{P}\left[Z_{j}=1|Y_{j}=1\right]={\eta_{1}}^{I\left({b_{j}}^{*}=1\right)}\lambda {\left(\alpha ;{b_{j}}^{*}\right)}^{I\left({b_{j}}^{*}>1\right)},$$

Where $\lambda \left(\alpha ;{b_{j}}^{*}\right)=1-\frac{1}{1+\exp\left(\alpha_{0}+\alpha_{1}{b_{j}}^{*}+\alpha_{2}{b_{j}}^{*2}\right)}$ and *η_1 _*is an unknown constant, which is not of our interest. The b_j_* is defined using the metabolite in reference:

(5) $${b_{j}}^{*}=\underset{k\in C,I\left(r_{kj}<h\right)}{\sum }1/a_{k}.$$

where b_j_* includes metabolite *j *itself as a neighbor to account for the fact that *Y_j _*= 1.

**Layer 3: **For reference metabolites matched to at least one target metabolite, we consider conditional probability of *W_jl _*given *Y_j _*and *Z_j_*, the correctness of those matches. For those matches of metabolite *j *with *Y_j _*= 1 and *Z_j _*= 1, we consider the following model:

(6) $$\text{P}\left(W_{jl}=1|Y_{j}=1,\;Z_{j}=1\right)=\tau .$$

Since is between 0 and 1, this implies that our matching is not always correct even though metabolite *j *is true positive.

**Layer 4: **we use a mixture model to characterize the distribution of the mixture similarity scores:

(7) $$f\left(S_{j}|W_{j}\right)=\underset{l}{\prod }f_{T}{\left(S_{jl};\phi_{T}\right)}^{W_{jl}}f_{F}{\left(S_{jl};\phi_{F}\right)}^{\left(1-W_{jl}\right)},$$

where *f *is the mixture of densities *f_T _*and *f_F _*that are the distributions of the scores of the correct matches and incorrect matches, respectively, and *ϕ_T _*and *ϕ_F _*are corresponding parameters.

**Rationale behind the model: **the rationale behind the use of a logistic function in layer 2 results from logistic regression. In other words, we investigated the relationship between *Z *and corresponding competition scores by logistic regression. Then, we found that quadratic function is statistically significant (see Section 1-3 in Additional file 1). Note that score function (*f*) consists of two score density functions: *f *= π*f_T _*+ (1-π)*f_F. _*According to the distribution of observed scores, the specification of the score functions is decided. Here we assume normality. According to the distribution of observed scores, either *f_T _*or *f_F _*could be assumed a normal mixture. The parameter vector to be estimated is $\theta =\left(\rho ,\;\tau ,\;\alpha_{0},\;\alpha_{1},\;\alpha_{2},\;\beta_{0},\;\beta_{1},\;\beta_{2},\;\mu_{T},\;\sigma_{T}^{2},\;\mu_{F},\;\sigma_{F}^{2},\;\pi_{1},\;\pi_{2}\right).$ More details about model description can be found in [11].

### Matching Confidence

The matching confidence of metabolite *j *in reference to a target metabolite can be calculated as the posterior probability of *W_jl_*:

(8) $${\text{P}}_{jl}=\text{P}\left[W_{jl}=1|Z_{j}=1,\;S_{j};\hat{\theta }\right]$$

where $\hat{\theta }$ is the estimated parameter vector. Note that this matching confidence plays a key role in the first step of peak alignment procedure.

Since we treat *Y *and *W *as the unobserved variables, we employ Expectation-Maximization (EM) algorithm to handle such latent variables [12]. More details about EM are provided in Additional file 1.

### Mixture similarity score

Mixture similarity score is defined as:

(9) $$S\left(A,\;B\right)=w\frac{D}{1+D}+\left(1-w\right)C/90,$$

where *S*(*A*, *B*) is mixture similarity score between two peaks A and B. Note that *w *is weight (0≤*w*≤1), *D *is rank distance based on retention time, and *C *is cosine score, angle between two peaks in high dimensional space (see Additional file 1 for details). Clearly, there is unit imbalance between RT distance and spectrum similarity. To balance them, we rescale rank distance (*D*) and angle (*C*) in Equation (9). Since considering rank of RT as a measure of closeness in RT reduces false positive rate [5], we take into account the elution order of RT in Equation (9). When calculating similarity score, we prefer small value of *w*(0 ≤ *w *≤ 0.5) in order for spectrum similarity to play a important role in similarity score.

### Peak alignment algorithm

**Figure 7:**
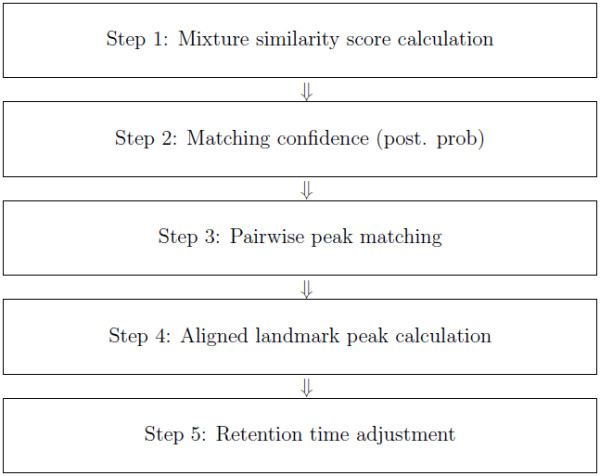
**Schematic representation of peak alignment algorithm**. Five key steps of peak alignment algorithm.

As aforementioned, our algorithm for peak alignment consists of two steps: pairwise peak matching and retention time adjustment. The process of our algorithm is summarized in Figure 7.

#### Pairwise peak matching

**Figure 8:**
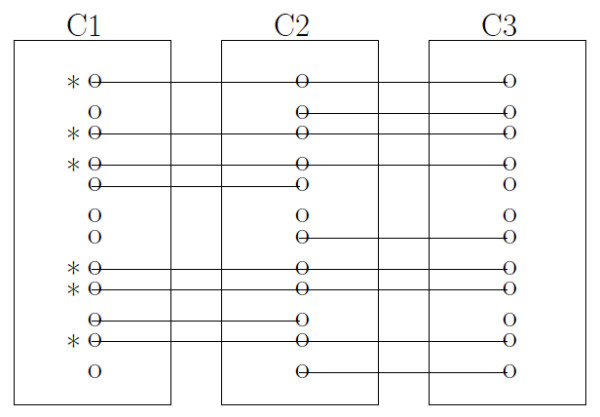
**An illustrative example of landmark peak selection**. 3 experiment outputs: C1, C2, and C3. Six peaks denoted by * are selected as landmark peaks. o presents compound within each output and - presents matching pairs with matching confidence greater than pre-specified cutoff value.

Given two experiment outputs, we calculate peak matching confidence through empirical Bayes model. Once the same calculation is done for all pairwise outputs, we then select peaks connecting through all outputs, which are called landmark peaks. As an illustration, suppose that we have 3 experiment outputs: C1, C2, and C3. In Figure 8, "o" presents metabolites within each experimental output and a connection line presents metabolite pairs with matching confidence greater than pre-specified cutoff value. The landmark peaks denoted by * are selected because those peaks exist in all outputs. We define *representative landmark peaks *as an average of those retention times (RT) across experiments.

#### Retention time adjustment

**Figure 9:**
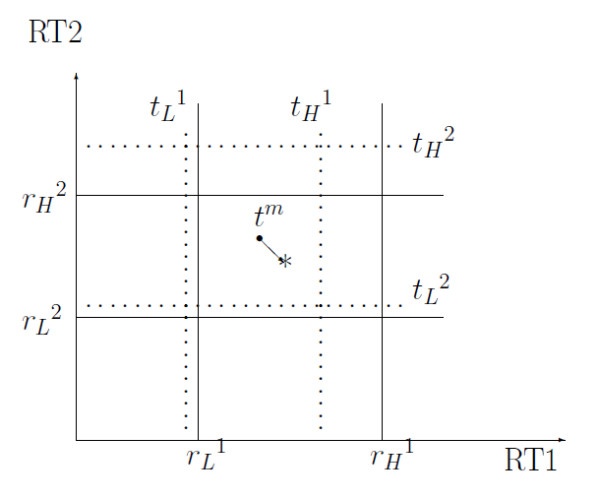
**A graphical representation of retention time adjustment**. A target peak (*t*^*m *^denoted by ·) to be aligned is moved to new position denoted by (*) after peak alignment. X-axis presents the first retention time and Y-axis presents the second retention time. The dotted grid presents target retention time (*t_L_*^1 ^and *t_H_*^1^: the closest target RT1 below and above *t*^*m*^, *t_L_*^2 ^and *t_H_*^2^: the closet target RT2 below and above *t*^*m*^). The solid grid presents reference retention time (*r_L_*^1 ^and *r_H_*^1^: the corresponding reference RT1 aligned to target RT1, *r_L_*^2 ^and *r_H_*^2^: the corresponding reference RT2 aligned to target RT2).

Given representative landmark peaks, we align all peaks in each experiment output with respect to them sequentially in both dimensions (especially, lattice-wise, not peak-wise). For example, a target peak to be aligned (*t*^m ^denoted by • in Figure 9) is moved to * after RT adjustment. X-axis presents the first dimensional retention time and Y-axis presents the second dimensional retention time. The dotted grid presents target retention time (*t_L_*^1 ^and *t_H_*^1^: the closest target RT1 below and above *t*^m^, *t_L_*^2 ^and *t_H_^2^*: the closest target RT2 below and above *t^m^*). The solid grid presents reference retention time (*r_L_*^1 ^and *r_H_*^1^: the corresponding reference RT1 aligned to target RT1, *r_L_*^2 ^and *r_H_*^2^: the corresponding reference RT2 aligned to target RT2). The mathematical formula for the first dimensional retention time of new aligned peak (*) is given:

(10) $$RT1^{new}={r_{L}}^{1}+\Delta \left({r_{H}}^{1}-{r_{L}}^{1}\right)$$

Where $\Delta =\frac{{t_{m}}^{1}-{t_{L}}^{1}}{{t_{H}}^{1}-{t_{L}}^{1}}.$ Similarly, new value for the second dimensional retention time can be obtained. In summary, retention time adjustment process consists of two sequential steps: the first dimension RT adjustment and the second dimension RT adjustment. RTs for all peaks in each dimension are aligned simultaneously with respect to the reference lattice, which is constructed based on representative landmark peaks. More details are provided in Additional file 1.

## Authors' contributions

JJ and CS designed and formulated the statistical model. JJ developed the programs to implement the model. XZ and XS designed the two experiments. XS conducted the experiments. JJ and SK conceived the study and conducted comparison study. All authors read and approved the final manuscript.

## Supplementary Material

Additional file 1 **supplementary materials**. This file include formula derivation and some results including tables and plots. Click here for file
