# Supplementary material for: Model-based peak alignment of metabolomic profiling from comprehensive two-dimensional gas chromatography mass spectrometry
Source: BMC Bioinformatics. 2012 Feb 8;13:27. doi: 10.1186/1471-2105-13-27 (PMC3323827; doi:10.1186/1471-2105-13-27)
Supplement: Additional file 1 — supplementary materials. This file include formula derivation and some results including tables and plots. [file 1471-2105-13-27-S1.PDF]

Supplementary Materials: Model-based peak alignment of metabolomic profiling from comprehensive two-dimensional gas chromatography mass spectrometry

1 EM and KDE

1.1 E-step

In the E-step, we deal with missing variables through conditional expectation of complete-data log likelihood given observed data, which produces the target function $Q(\theta;\theta^{(k)})$. The target function is defined by:

$$Q(\theta;\theta^{(k)}) = E(l(\theta)|Z,S,\theta^{(k)}), \tag{1}$$

where $\theta = (\rho,\tau,\alpha,\beta,\phi_T,\phi_F)$ and $l(\theta) = \sum_{j=1}^N [Y_j \ln\rho + (1-\rho)\ln(1-\rho)] + \sum_{j=1}^N [Z_j \ln\{\lambda(\alpha;a_j)^{Y_j}\gamma(\beta;b_j)^{1-Y_j}\} + (1-Z_j)\ln\{(1-\lambda(\alpha;a_j))^{Y_j}(1-\gamma(\beta;b_j))^{1-Y_j}\}] + \sum_{j=1}^N \sum_{k=1}^{K_j} Y_j Z_j \{W_{jk}\ln\tau + (1-W_{jk})\ln(1-\tau)\} + \sum_{j=1}^N \sum_{k=1}^{K_j} \{W_{jk}\ln f_T(S_{jk};\phi_T) + (1-W_{jk})\ln f_F(S_{jk};\phi_F)\}$.
Actually, we need to calculate three different conditional expectations: $E(Y_j|Z_j,S_{jk},\theta^{(k)})$, $E(W_{jk}|Z_j,S_{jk},\theta^{(k)})$ and $E(Y_j W_{jk}|Z_j,S_{jk},\theta^{(k)})$.

1.2 M-step

Here we maximize the target function given in the E-step with respect to parameters, i.e., we have to solve a system of estimating equations. Since we denote the λ and γ as logistic functions and logistic function is not linear, parameters like α and β have nonlinear estimating equation while other parameters have linear one and their estimators exist in closed form. To solve such nonlinear estimating equations for α and β, general-purpose optimization based on quasi Newton method is used (Broyden70,Fletcher70,Goldfarb70,Shanno70).

Estimators with explicit formula are represented by:

$$\hat{\rho} = \frac{1}{N}\sum_{j=1}^N E[Y_j|Z_j,S_j],$$

$$\hat{\tau} = \frac{1}{\sum_j\sum_k Z_j E[Y_j|Z_j]}\sum_j\sum_k Z_j E[Y_j W_{jk}|Z_j,S_j],$$

$$\hat{\mu}_T = \frac{1}{\sum_j\sum_k E[W_{jk}|Z_j,S_{jk}]}\sum_j\sum_k S_{jk}E[W_{jk}|Z_j,S_j],$$

$$\hat{\sigma_T}^2 = \frac{1}{\sum_j\sum_k E[W_{jk}|Z_j,S_{jk}]}\sum_j\sum_k (S_{jk}-\hat{\mu}_T)^2 E[W_{jk}|Z_j,S_j],$$

$$\hat{\mu}_F = \frac{1}{NF} \sum_j \sum_k S_{jk}(1 - E[W_{jk}|Z_j, S_j]),$$

$$\hat{\sigma_F}^2 = \frac{1}{NF} \sum_j \sum_k (S_{jk} - \hat{\mu}_F)^2 (1 - E[W_{jk}|Z_j, S_j])$$

where $NF = \sum_j \sum_k (1 - E[W_{jk}|Z_j, S_{jk}])$.

1.3 Rationale behind the model

As mentioned in main text, the rationale behind the use of a logistic function in layer 2 results from logistic regression. In other words, we investigated the relationship between Z and corresponding competition scores by logistic regression to get some sense of functions in layer 2 (γ and λ). We did logistic regression with real biological data by using the built-in function in statistical software R. Then, we found that quadratic function is statistically significant in case of function λ at the significant level of 0.05. Analysis results (p values) are summarized in Table S1. On the other hand, even though the quadratic term

Table 1: Summary of logistic regression results (p value)

	$\gamma(b_j)$	$\lambda(b_j^*)$
linear	0.01	0.001
quadratic	(0.014, 0.572)	(0.001, 0.019)

in case of function γ is not statistically significant, the quadratic term coupled with linear term is helpful to explain the relationship.

1.4 Kernel density estimator

When we want to know the distribution information of data, the simplest way is to use histogram. However it has many drawbacks including: (1)it depends heavily on bin size and its starting point (2)it is not continuous at all bin starting and end points. That's why kernel density estimator (KDE), which can be considered as a continuous version of histogram, has been widely used instead. The definition of KDE is given by

$$\hat{f}(x) = \frac{1}{nh} \sum_{i=1}^{n} K\left(\frac{x - x_i}{h}\right)$$

where h is a bandwidth, K is a kernel function and n is the number of sample. In our analysis, we used normal kernel.

2 Experimental details

2.1 Experiment I: mixture of compound standards

A mixture of 35 amino acids, fatty acids and organic acids were prepared in pyridine. The concentration of each acid in the mixture was 1 mg/mL. A 50 μL aliquot of the mixture was derivatized with 100 μL of N-Methyl-N-(Tert-Butyldimethylsilyl)trifluoroacetamide (MTBSTFA) for 30 min at 60^oC. All GCxGC/TOF-MS analyses were performed on a LECO Pegasus 4D time-of-flight mass spectrometer (TOF-MS) with a Gerstel MPS2 auto-sampler. The Pegasus 4D GCxGC/TOF-MS instrument was equipped with an Agilent 6890 gas chromatograph featuring a LECO two stage cryogenic modulator and secondary oven. A $30m \times 0.25mm\ id. \times 0.25\mu m$ film thickness, Rxi-5ms GC capillary column was used as the primary column for the GCxGC/TOF-MS analysis. A second GC column of $2m \times 0.10mm\ id. \times 0.10\mu m$ film thickness, BPX-50 was placed inside the secondary GC oven after the thermal modulator. The helium carrier gas flow rate was set to 1.0 mL/min at a corrected constant flow via pressure ramps. A $2\mu L$ liquid sample was injected into the liner using the splitless mode with the injection port temperature set at 260^oC. The first-dimension column oven ramp began at 60^oC with a 0.5-min hold after which the temperature was programmed to 280^oC at a rate of $8^oC/min$ and then held at this temperature for 6 min. The second-dimension column temperature was maintained 5^oC higher than the corresponding first-dimension column. The programming rate and hold times were the same for the two columns. The thermal modulator was set to $+20^oC$ relative to the primary oven and a modulation time of 5 s was used. The MS mass range was $45 - 750m/z$ with an acquisition rate of 200 spectra per second. A 700 s solvent delay was used. The ion source chamber was set at 230^oC with the MS transfer line temperature set to 260^oC and the detector voltage was $1800V$ with an electron energy of $70eV$. The LECO ChromaTOF software version 3.41 equipped with the National Institute of Standards and Technology (NIST) MS database (NIST MS Search 2.0, NIST/EPA/NIH Mass Spectral Library; NIST 2002) was used for instrument control, spectrum deconvolution and metabolite identification.

2.2 Experiment II: rat plasma

Metabolites were extracted from a $100\mu L$ rat plasma sample using $900\mu L$ of organic solvent mixture (methanol:water = 8:1). A $50\mu L$ aliquot of plasma extract were further derivatized with N-tert-Butyldimethylsilyl-N-methyltrifluoroacetamide (MTBSTFA). The derivatized metabolite extract was spiked at a concentration of $2.5\mu g/mL$ with a deuterated six component semi-volatiles internal standard (ISTD) mixture prior to GCxGC/TOF-MS analysis by a LECO Pegasus 4D time-of-flight mass spectrometer (TOF-MS). A $30m \times 0.25mm\ id. \times 0.25\mu m$ film thickness, Rxi-5ms, GC capillary column was used as the primary column for the GCxGC/TOF-MS analysis. In the GCxGC configuration, a second column $1.2m \times 0.10mm\ id. \times 0.10\mu m$ film thickness, BPX-50, was placed inside

the LECO secondary GC oven after the thermal modulator. Helium carrier gas flow rate was set to $1.0mL$ per minute at a corrected constant flow via pressure ramps. A $1\mu L$ splitless liquid injection was made with the injection port temperature set at 260^oC. The primary column was programmed with an initial temperature of 60^oC for 0.5 minute and then ramped at 7^oC per minute to 315^oC for 8.5 minutes. The secondary column temperature program was set to an initial temperature of 65^oC for 0.5 minute and then ramped at 7^oC per minute to 320^oC with an 8.5 minutes hold time for a total runtime of 45.43 minutes. The thermal modulator was set to $+20^oC$ relative to the primary oven and a modulation time of 5 seconds was used. The MS mass range was $10-750m/z$ with an acquisition rate of 150 spectra per second. The ion source chamber was set at 230^oC with the MS transfer line temperature set to 260^oC and the detector voltage was $1800V$ with an electron energy of $-70eV$. The acquired data was processed with a user defined data processing method. The LECO ChromaTOF software version 3.41 equipped with the National Institute of Standards and Technology (NIST) MS database (NIST MS Search 2.0, NIST/EPA/NIH Mass Spectral Library; NIST 2002) was used for instrument control, spectrum deconvolution and metabolite identification.

3 Algorithm

3.1 Peak alignment

Once representative landmark peaks are given, we make grid net using the retention times of those peaks. Since peak retention time can be inside or outside landmark peak grid, we need to define peak alignment formula in three different ways. The formula for the peaks between the smallest and the largest landmark peak retention time (say inside peak) is defined by

$$RT_t{}^{new} = RT_r{}^L + \Delta(RT_r{}^H - RT_r{}^L), \tag{2}$$

where $RT_r{}^L$ and $RT_r{}^H$ are high and low reference retention time, $\Delta = \frac{RT_t{}^A - RT_t{}^L}{RT_t{}^H - RT_t{}^L}$, and $RT_t{}^A$ is peak retention time to be aligned.

The formula for the peaks with RT smaller than the smallest landmark peak RT is defined by

$$RT_t{}^{new} = RT_r{}^H - \Delta(RT_r{}^H - RT_r{}^L), \tag{3}$$

where $RT_r{}^L$ and $RT_r{}^H$ are the smallest and the second smallest reference retention time, $\Delta = \frac{RT_t{}^H - RT_t{}^A}{RT_t{}^H - RT_t{}^L}$, and $RT_t{}^A$ is peak retention time to be aligned.

The formula for the peaks with RT larger than the largest landmark peak RT is defined by

$$RT_t{}^{new} = RT_r{}^L + \Delta(RT_r{}^H - RT_r{}^L), \tag{4}$$

where $RT_r{}^H$ and $RT_r{}^L$ are the largest and the second largest reference retention time, $\Delta = \frac{RT_t{}^A - RT_t{}^H}{RT_t{}^H - RT_t{}^L}$, and $RT_t{}^A$ is peak retention time to be aligned.

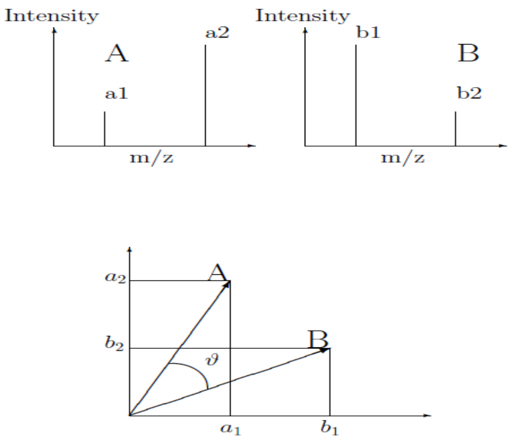

Figure 1: Geometric representation of cosine score in two-dimensional space.

4 Matching Results

4.1 Mixture similarity score and matching accuracy measure

Mixture similarity score is defined as:

$$S(A, B) = w\frac{D}{1 + D} + (1 - w)C/90, \tag{5}$$

where w is weight, D is rank distance based on retention time, and C is cosine score. As an illustration of cosine score, the geometric representation of scaled dot product (=cos(ϑ)) in two-dimensional space is given in Figure S1. To calculate the rank distance D, we first consider the sum of two dimensional retention times of each compound and assign corresponding rank to each compound. We then calculate the rank difference in paired experiment output. Clearly, there is unit imbalance between RT distance and spectrum similarity. To balance them, we rescale rank distance (D) and angle (C) so they have same range. Through tuning parameter (w), we control the contribution of rank distance and cosine score.

As a measure for matching accuracy, we consider three different measures: positive predictive value (PPV), true predictive value (TPV) and F1 value, which is harmonic mean of PPV and TPV. Such standard comparison measures can be easily obtained by using 2×2 table (Table S2). For clarity, PPV, TPV, and F1 are defined as follows:

$$PPV = \frac{C11}{C11 + C12}, \tag{6}$$

$$TPV = \frac{C11}{C11 + C21}, \tag{7}$$

Table 2: 2×2 table: Test outcome is based on the results by any method

		Gold standard	
		Positive	Negative
Test outcome	Positive	C11	C12
	Negative	C21	C22

$$F1 = \frac{2PPV \times TPV}{PPV + TPV}. \quad (8)$$

4.2 Results summary

Experiment I, a mixture of compound standards, has three datasets obtained by different experimental setup, i.e., different gradient temperature: 5^oC, $7^oC, 10^oC$ called dataset1, dataset2, dataset3, respectively. Using these three homogeneous datasets, we produce a heterogeneous dataset, which is called dataset4. That is, each dataset has 10, 2, 4 and 3 replicates. We consider four weight parameter($w = 0.1, 0.2, 0.3, 0.5$) and five different cutoff values (cutoff=0.6,0.7,0.8,0.9,0.95). For each combination of parameters, we calculated sensitivity (TPV), positive predictive values (PPV) and F1. Then we selected the best results in terms of F1 score. Table S3 summarizes the results. Also, we provide a table summarizing DISCO results (Table S4).

Here we provide a table (Table S5) including standard error corresponding to Table 3 in main body: standard error for dataset1, dataset4 and Experiment II.

4.3 DISCO

In Table S6, we provide results obtained by DISCO: TPV, PPV and F1 score.

4.4 Venn Diagram corresponding to best F1

We compare our matching results with the results from ChromaTOF software. To do that, we selected the best pair from each dataset. Best pair means a pair with the best F1 score. In dataset1, ${R_8}^5$ and ${R_9}^5$ ($w = 0.2$ and cuoff=0.8) were selected. In dataset2, ${R_1}^7$ and ${R_2}^7$ ($w = 0.3$ and cuoff=0.6) were selected and ${R_3}^{10}$ and ${R_4}^{10}$ ($w = 0.1$ and cuoff=0.6) in dataset3. For heterogeneous case, ${R_1}^5$ and ${R_1}^7$ ($w = 0.1$ and cuoff=0.6) were selected. In addition, from Experiment II, D_3 and D_4 ($w = 0.1$ and cuoff=0.8) were selected. Venn diagrams corresponding to the selected pairs are given in Figure S2:

The Venn diagram (Figure S2) can be summarized more concisely in Table S7:

Table 3: Our method: Sensitivity, PPV and F1 values

Dataset1	cutoff=0.7	cutoff=0.8	cutoff=0.9	cutoff=0.95
w=0.1	0.918/0.931/0.924	0.918/0.931/0.924	0.904/0.943/0.923	0.930/0.930/0.930
w=0.2	0.918/0.931/0.924	0.918/0.944/0.931	0.904/0.943/0.923	0.930/0.930/0.930
w=0.3	0.918/0.931/0.924	0.918/0.931/0.924	0.904/0.943/0.923	0.930/0.930/0.930
w=0.5	0.918/0.931/0.924	0.918/0.931/0.924	0.904/0.943/0.923	0.930/0.930/0.930
Dataset2	cutoff=0.7	cutoff=0.8	cutoff=0.9	cutoff=0.95
w=0.1	0.778/0.933/0.848	0.778/0.933/0.848	0.708/0.927/0.803	0.639/0.920/0.754
w=0.2	0.778/0.933/0.848	0.764/0.932/0.840	0.708/0.927/0.803	0.611/0.917/0.733
w=0.3	0.778/0.933/0.848	0.764/0.932/0.840	0.708/0.927/0.803	0.583/0.913/0.712
w=0.5	0.778/0.949/0.855	0.764/0.948/0.846	0.708/0.944/0.810	0.611/0.936/0.739
Dataset3	cutoff=0.7	cutoff=0.8	cutoff=0.9	cutoff=0.95
w=0.1	0.817/0.951/0.879	0.817/0.951/0.879	0.803/0.950/0.870	0.775/0.948/0.853
w=0.2	0.817/0.951/0.879	0.817/0.951/0.879	0.803/0.950/0.870	0.775/0.948/0.853
w=0.3	0.817/0.951/0.879	0.817/0.951/0.879	0.803/0.950/0.870	0.761/0.947/0.844
w=0.5	0.817/0.951/0.879	0.817/0.951/0.879	0.803/0.950/0.870	0.761/0.947/0.844
Dataset4	cutoff=0.7	cutoff=0.8	cutoff=0.9	cutoff=0.95
w=0.1	0.833/0.882/0.857	0.819/0.881/0.849	0.819/0.881/0.849	0.792/0.877/0.832
w=0.2	0.833/0.882/0.857	0.819/0.881/0.849	0.806/0.879/0.841	0.778/0.875/0.824
w=0.3	0.833/0.882/0.857	0.819/0.881/0.849	0.819/0.881/0.849	0.792/0.877/0.832
w=0.5	0.833/0.882/0.857	0.819/0.881/0.849	0.819/0.881/0.849	0.792/0.877/0.832
Rat	cutoff=0.7	cutoff=0.8	cutoff=0.9	cutoff=0.95
w=0.1	0.758/0.516/0.614	0.730/0.537/0.619	0.653/0.570/0.609	0.536/0.689/0.603
w=0.2	0.746/0.518/0.612	0.722/0.536/0.615	0.661/0.582/0.619	0.504/0.635/0.562
w=0.3	0.750/0.517/0.612	0.714/0.532/0.609	0.637/0.568/0.601	0.536/0.630/0.580
w=0.5	0.750/0.517/0.612	0.722/0.539/0.617	0.653/0.572/0.610	0.508/0.660/0.574

Table 4: DISCO: Sensitivity, PPV and F1 values

Dataset	cutoff=0.7	cutoff=0.8	cutoff=0.9	cutoff=0.95
dataset1	0.873/1.000/0.932	0.873/1.000/0.932	0.873/1.000/0.932	0.877/0.985/0.928
dataset2	0.847/1.000/0.917	0.833/1.000/0.909	0.819/1.000/0.901	0.833/1.000/0.909
dataset3	0.743/0.963/0.839	0.743/0.963/0.839	0.743/0.945/0.832	0.718/0.944/0.816
dataset4	0.694/0.943/0.800	0.694/0.943/0.800	0.694/0.943/0.800	0.694/0.962/0.806
rat	0.431/0.723/0.540	0.427/0.711/0.534	0.431/0.718/0.539	0.415/0.746/0.534

4.5 Manual validation

We manually checked the peak lists obtained by our method, but not identified from ChromaTOF for homogeneous data from Experiment I (dataset 1). The

Table 5: Summary of averaged best F1 values

Method	F1	dataset1	dataset4	rat
DISCO	mean/se	0.902/0.011	0.781/0.025	0.496/0.020
OUR	mean/se	0.878/0.021	0.839/0.015	0.567/0.018

Table 6: Summary of DISCO results

rho	TPV	PPV	F1
0.1	0.7930	0.9653	0.8701
0.2	0.7988	0.9683	0.8749
0.3	0.8006	0.9695	0.8765
0.4	0.8000	0.9695	0.8761
0.5	0.8001	0.9710	0.8767
0.6	0.8013	0.9717	0.8776
0.7	0.8010	0.9717	0.8775
0.8	0.8005	0.9732	0.8777
0.9	0.7928	0.9745	0.8734
0.93	0.7900	0.9744	0.8717
0.95	0.7888	0.9763	0.8717
0.97	0.7802	0.9775	0.8670
0.99	0.7466	0.9802	0.8464

Table 7: Alignment results by both methods

dataset (selected pair)	DISCO only	Common area	EBM only
dataset1 (${R_8}^5, {R_9}^5$)	6	67	4
dataset2 (${R_1}^7, {R_2}^7$)	14	58	4
dataset3 (${R_3}^{10}, {R_4}^{10}$)	13	58	3
dataset4 (${R_1}^5, {R_1}^7$)	12	60	8
Experiment II (D_3, D_4)	67	181	156

corresponding Venn diagram is provided in Figure S2 (see the top left plot). We then found that one of the four peak pairs was aligned correctly by our method (the first row in Table S8) while the other three might not be correct. Also, we checked six peak pairs, which have same compound name by ChromaTOF, but not aligned by our method. Among them, ChromaTOF made wrong decision for three peak pairs. In other words, those three pairs cannot be aligned correctly through the use of the names given by ChromaTOF (see rows 5, 6 and 7 in Table S8). Although the three peak pairs are verified to be incorrect by man-

ual examination, we can also imagine by our method that those pairs are not correctly aligned based on similarity scores corresponding to the pairs (33.74, 33.14 and 51.78 in Table S8). These manual checking demonstrates that our alignment can be used to improve the accuracy of the compound identification.

We further investigated the relationship between alignment results by two methods. For this, we selected 2 aligned peak pairs in sample: CAS 193-39-5 and 629-92-5. Our method assigned a compound with CAS 193-39-5 in sample to a compound with CAS 191-24-2 in library because the peak pair has the best mixture similarity score (9.67). On the other hand, gold standard (GS) assigned the sample compound (193-39-5) to the compound with same CAS in reference. As we can see in Table S8, this peak pair has similarity score of 9.69, which is very close to 9.67. Actually, it was the second best by our scoring system. For another compound (CAS: 629-92-5) in target peak list, we can see the same situation. Based on our score, the compound was assigned to a compound with CAS: 112-95-8 in library because their similarity score is the best (1.36). However, the score difference from the second best (1.63) is very small (i.e.,1.63-1.36=0.27).

Table 8: Manual check: 4 peak pairs and 6 peak pairs

Sample CAS	library CAS	score
106-44-5	108-39-4	3.24
91-57-6	90-12-0	1.44
629-92-5*	112-95-8	1.36
193-39-5*	191-24-2	9.67
95-50-1	95-50-1	37.74
87-86-5	87-86-5	33.14
935-95-5	935-95-5	51.78
193-39-5*	193-39-5	9.69
111-65-9	111-65-9	13.15
629-92-5*	629-92-5	1.63

We provide two raw data plots used for manual inspection. The Figure S3 corresponds to the peak pair (the first row in Table S8), which is the case that our method is correct. As aforementioned, even though they have different name given by ChromaTOF, they are correctly aligned by our method, implying that ChromaTOF assigned a wrong name.

Figure S4 presents the raw data plot, which correspond to the peak pair with the same compound name (CAS: 95-50-1), but not aligned by our method.

5 Alignment Results

For peak alignment, we need to select landmark peaks, which are used for retention time correction. For this, we use threshold ($h = 40$) and weight ($w = 0.1$) for mixture score and apply cutoff value of 0.9 to posterior probability. Then we got 11, 40, 28, and 24 landmark peaks from Experiment I and 31 landmark peaks from Experiment II. KDE plots before/after RT adjustment for dataset1 and dataset2 are given in Figure S5. The corresponding KDE plots for other datasets such as dataset3 and dataset4 are given in main text (Figures 2 and 3).

For two dimensional view on alignment results (say joint view), scatter plots of RT before/after RT adjustment for dataset4 are given in Figure S6. Similarly, scatter plots of RT before/after RT adjustment for Experiment 2 are given in Figure S7.

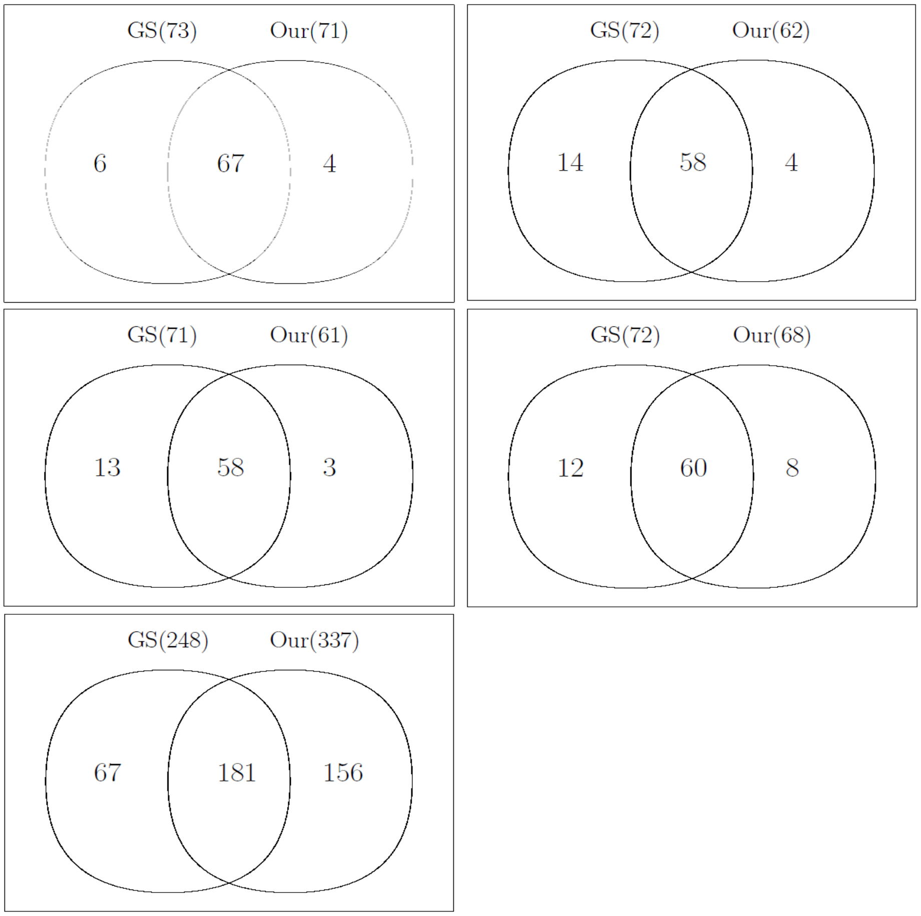

Figure 2: Venn diagram corresponding to best F1 measure (homogeneous: dataset1, dataset2, dataset3; heterogeneous: dataset4). Top: dataset1 (left) and dataset2 (right). Middle: dataset3 (left) and dataset4 (right). Bottom: Experiment2

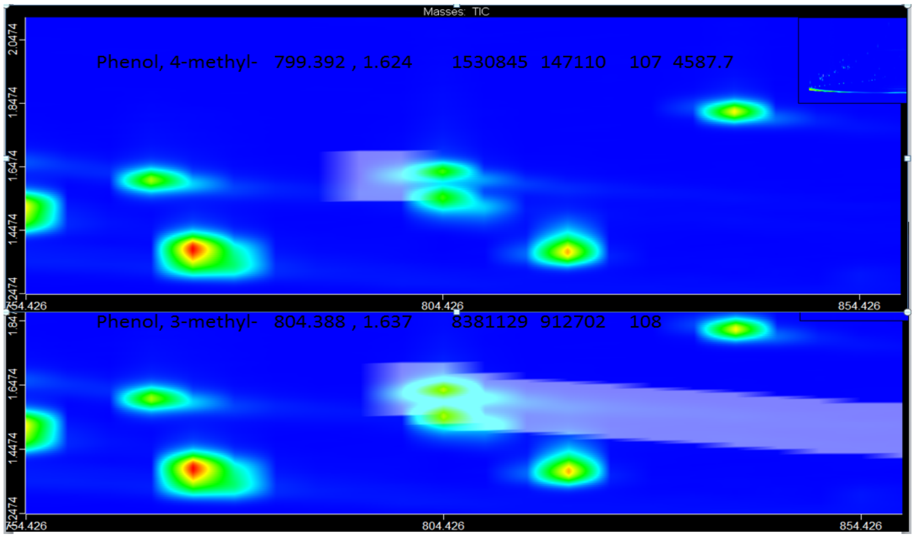

Figure 3: Raw data plot; Top: raw data plot for compound with CAS 106-44-5 in sample R_9^5; Bottom: raw data plot for compound with CAS 108-39-4 in sample R_8^5

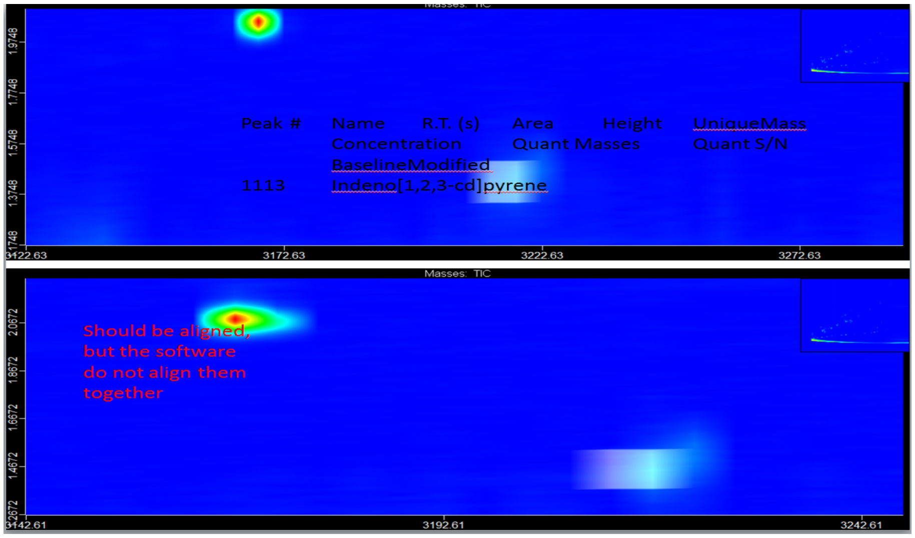

Figure 4: Raw data plot for compound with CAS 193-39-5 in both R_9^5 and R_8^5

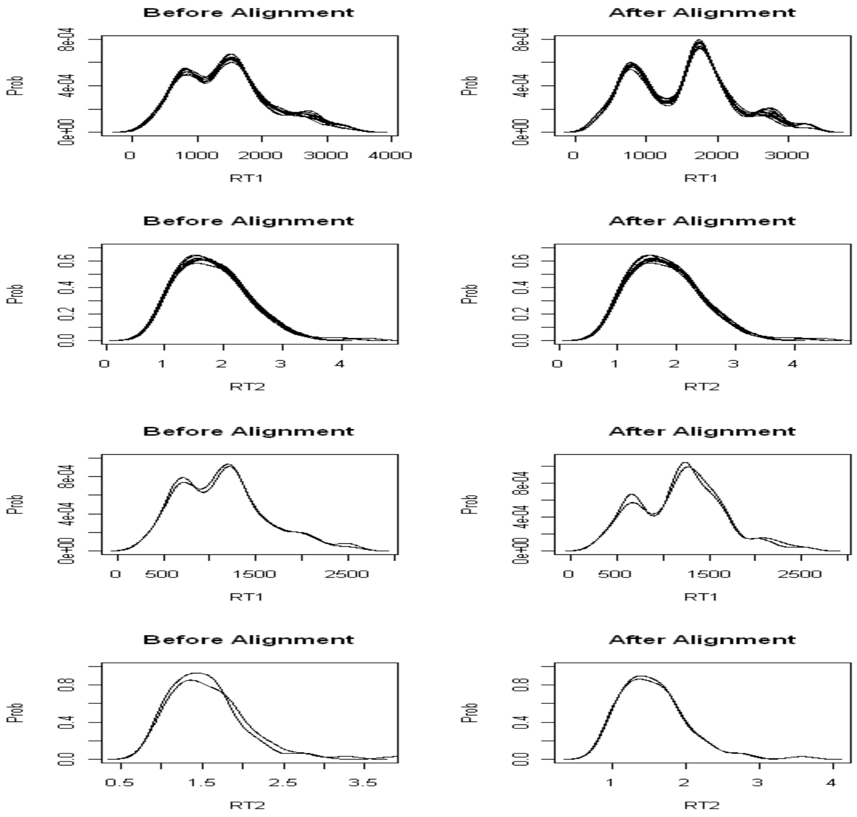

Figure 5: Experiment1: KDE plots before/after RT adjustment for datasets 1 and 2. Top: 10 curves for dataset1. Bottom: 2 curves for dataset2

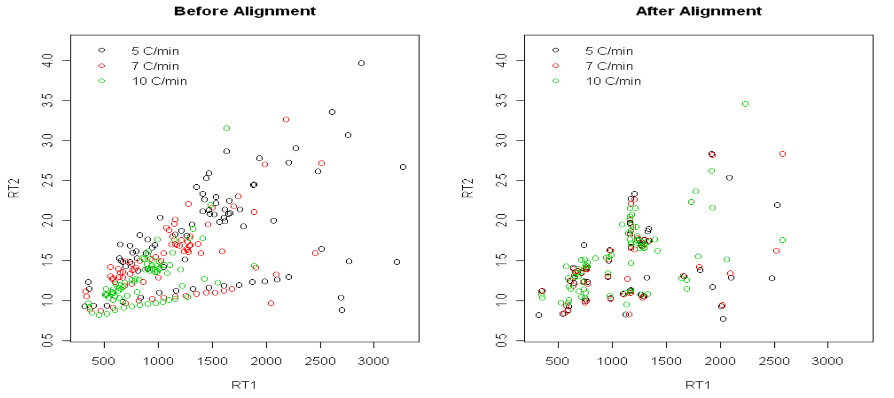

Figure 6: Experiment1(dataset4): scatter plots before/after RT adjustment for dataset4.

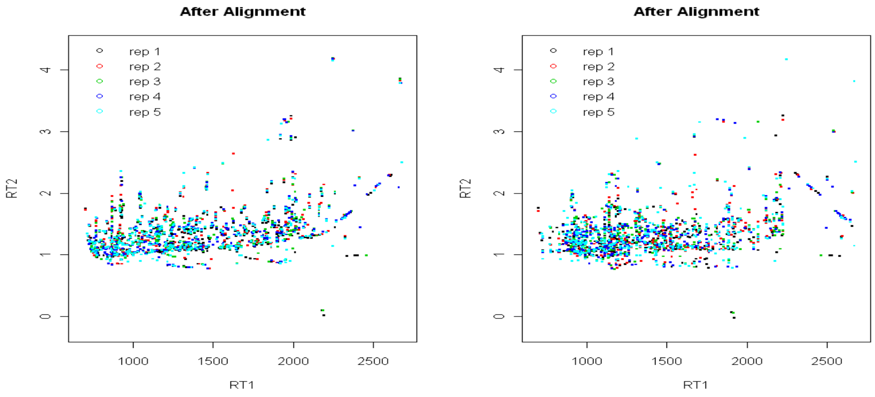

Figure 7: Experiment2: scatter plots before/after RT adjustment.
